# Pulmonary Tuberculosis: The Great Mimicker of Lung Cancer

**DOI:** 10.1002/ccr3.71919

**Published:** 2026-01-22

**Authors:** Laura Corrales‐Diaz Pomatto‐Watson, Hershan Singh Johl, Karleen Meiklejohn

**Affiliations:** ^1^ School of Medicine University of California Davis Sacramento California USA; ^2^ Department of Internal Medicine University of California Davis Sacramento California USA; ^3^ Department of Pathology and Laboratory Medicine University of California Davis Sacramento California USA

**Keywords:** acid‐fast bacilli, lung cancer, pulmonary tuberculosis, radiological mass, tuberculoma

## Abstract

Though tuberculosis (TB) is an ancient pathogen, diagnosis remains challenging. Distinguishing pulmonary tuberculoma, a rare manifestation of TB, from lung cancer is critical. We report an 85‐year‐old woman with a smoking history and TB‐endemic exposure, presenting with fatigue and anorexia. Imaging revealed a solitary pulmonary nodule (SPN), with biopsy confirming TB.

## Graphical abstract

Computed tomography (CT) scan of the chest with contrast indicating a soft tissue density in the left upper lobe.

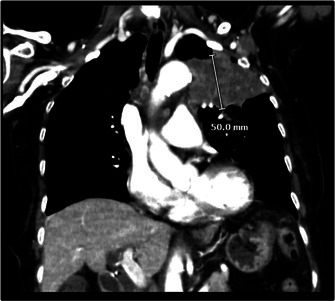

## 1 Introduction

*Mycobacterium tuberculosis* , the causative pathogen of TB, has maintained its stronghold as the leading single‐pathogen cause of global morbidity and mortality [1]. Despite the global impact, anti‐TB treatment has remained relatively unchanged for the past 50 years [2]. Largely attributed to its slow growth rate and multiple protective mechanisms, including an organismal efflux pump and the host's own defensive formation of granulomatous encasement. Due to its slow‐onset smoldering presentation, TB is a great clinical mimicker and can easily be mistaken for lung cancer. In 2024, the World Health Organization (WHO) estimated yearly global incidence of eight million cases of symptomatic TB, resulting in 1.4 million deaths [3], with most cases concentrated in South‐East Asia and Africa. Reactivation of the disease accounts for approximately 80% of all active TB cases [4].

Though TB is the leading infectious cause of death, it is vastly overshadowed by the mortality associated with lung cancer, which is the leading cause of cancer‐related death [5]. Non‐small cell lung cancer (NSCLC) accounts for the majority (~85%) of lung cancer subtypes [6], with a high correlation to smoking history [7]. Treatment strategies and patient prognosis are highly dependent upon stage of detection: more advanced stages correspond to poorer patient prognosis [6].

TB and lung cancer, though common worldwide, require correct identification for effective treatment. Incorrect diagnosis and resultant treatment delay can have devastating consequences on patient outcomes. Yet vague, overlapping clinical presentations (unintentional weight loss, fatigue, cough, fever, night sweats) [8] and sometimes indistinguishable features on diagnostic imaging [9], make the prediction more akin to a coin toss. Laboratory abnormalities, similarly, do not provide clarity; both diseases can induce an anemia of chronic disease, with mild‐to‐moderate anemia being common (~61%) in both TB [10] and lung cancer [11]. Hyponatremia, secondary to syndrome of inappropriate antidiuretic hormone (SIADH), may also be evident in both etiologies [12, 13]. Thus, clinicians are reliant on traditional gold‐standard approaches to ensure correct diagnosis, which include such modalities as lung biopsy and sputum cultures [14], many of which are slow processes and can delay the initiation of treatment.

This case highlights the continued diagnostic challenges faced by many clinicians in distinguishing between two differing diseases (pulmonary infectious vs. neoplastic etiology) in the setting where time to treatment is essential for improved patient outcomes.

## 2 Case Presentation

### 2.1 History and Examination

We present a case of an 85‐year‐old Vietnamese‐speaking woman with a history of hypertension, gastroesophageal reflux disease (GERD), and osteoarthritis who presented to the emergency department with a ten‐day history of fatigue and lack of appetite. She endorsed cold‐like symptoms (poor appetite and sleep, fatigue, chills, and night sweats), but no chest pain, and further review of systems was unremarkable. She had a significant smoking history but had quit seven years prior and had emigrated from Vietnam. The patient's BCG vaccine status was unknown. Initial vitals were remarkable only for a 101.1F temperature; she maintained oxygen saturation on room air and had an unremarkable physical exam.

### 2.2 Laboratory and Radiological Findings

The initial clinical impression was a possible infection with an unknown source, so multiple laboratory and imaging modalities were initiated. Complete blood count (CBC) showed no leukocytosis, but mild normocytic anemia (10.4–11.4). Basic metabolic panel (BMP) showed only mild hyponatremia (127–135 mmol/L); creatinine and BUN remained within normal limits. Liver function testing showed no abnormalities. Urinalysis showed moderate leukocyte esterase and bacteria, with subsequent urine culture being positive for *E. coli* . Blood culture had no growth throughout hospitalizations.

The urinalysis findings raised concern for a urinary tract infection, so a CT abdomen/pelvis was performed. The CT indicated bilateral hydronephrosis and a distended urinary bladder, concerning for possible chronic bladder outlet obstruction. A urinary catheter was placed to relieve the obstruction, and she was started on empiric antibiotics (Augmentin 875 mg, twice daily).

**FIGURE 1:**
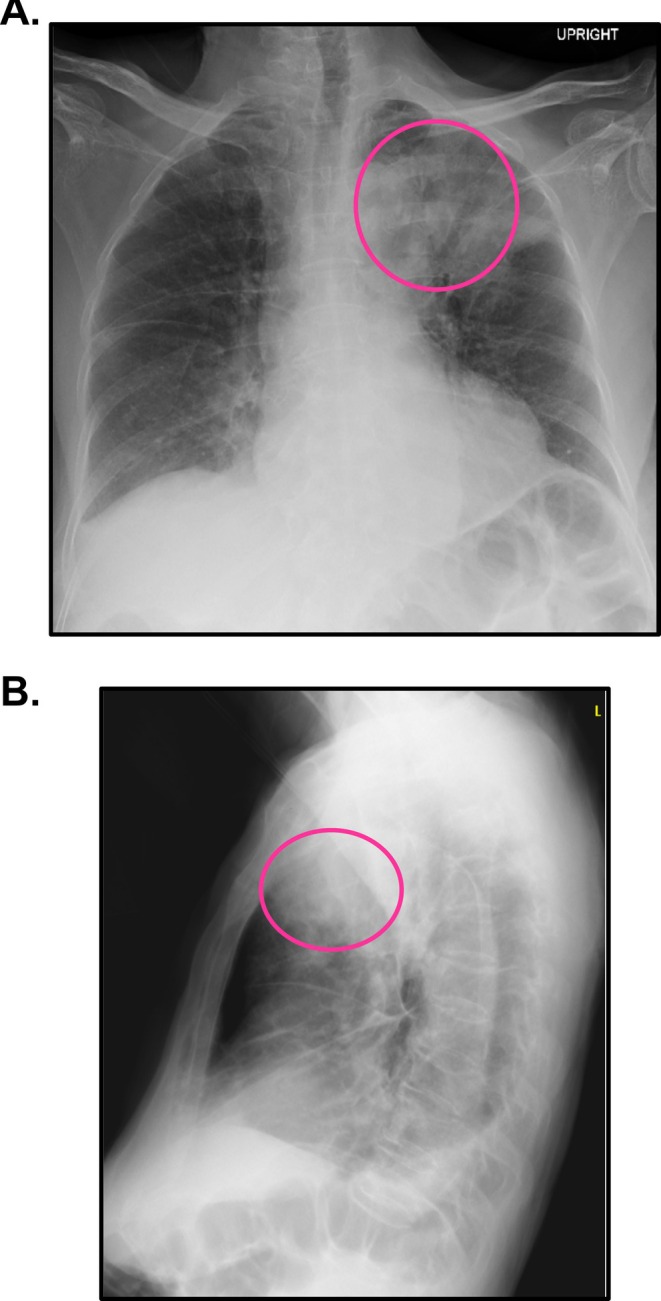
(A) Posterior–anterior (PA) and (B) lateral chest x‐ray indicating a left upper lobe lesion.

**FIGURE 2:**
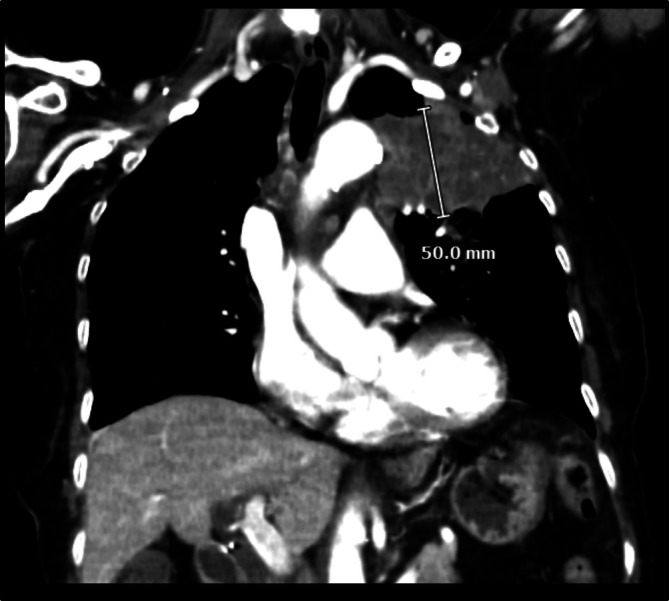
CT scan of the chest with contrast indicating a soft tissue density in the left upper lobe.

Initial concerns for infection also prompted a chest x‐ray (CXR) posterior–anterior (AP), which showed a mass in the left upper lobe of the lung (Figure 1A,B). Follow‐up CT‐chest with contrast showed a 6.3 × 7.6 × 5.0 cm left upper lobe mass with associated mediastinal lymphadenopathy (Figure 2). Though concerning for possible advanced malignancy [15], the CT was inconclusive in distinguishing an infectious consolidation from a neoplasm.

### 2.3 Clinical Differential Diagnoses and In‐Hospital Treatment

At this point in the patient's work up, the differential diagnoses included a malignant lung neoplasm, related to her extensive smoking history, or a possible infection, either acute (such as community‐acquired pneumonia (CAP)) or chronic, with possible reactivation of TB, given her migration from a TB‐endemic region [3]. CAP was less of a concern due to her unremarkable physical exam and relatively stable vitals; however, due to her age (> 65 years old) and increased risk of reduced immune response [16], she was treated with empiric antibiotics (addition of ceftriaxone 2 g and azithromycin 250 mg once per day) for possible CAP.

Additional diagnoses considered included autoimmune conditions, such as systemic lupus erythematous and giant cell arteritis, but given the lack of prior medical or family history of autoimmune diseases, and no evident skin rash or changes in vision, it was lower on the differential.

It is not possible to distinguish between active or latent TB by CXR [17], nor rule out a lung malignancy. The CT finding of a large mass with associated lymphadenopathy subsequently prompted additional work‐up: Bronchial sputum cultures were collected on three consecutive days, and a lung biopsy was collected for histopathologic evaluation and tissue culture. The patient was placed on TB‐isolation precautions.

**FIGURE 3:**
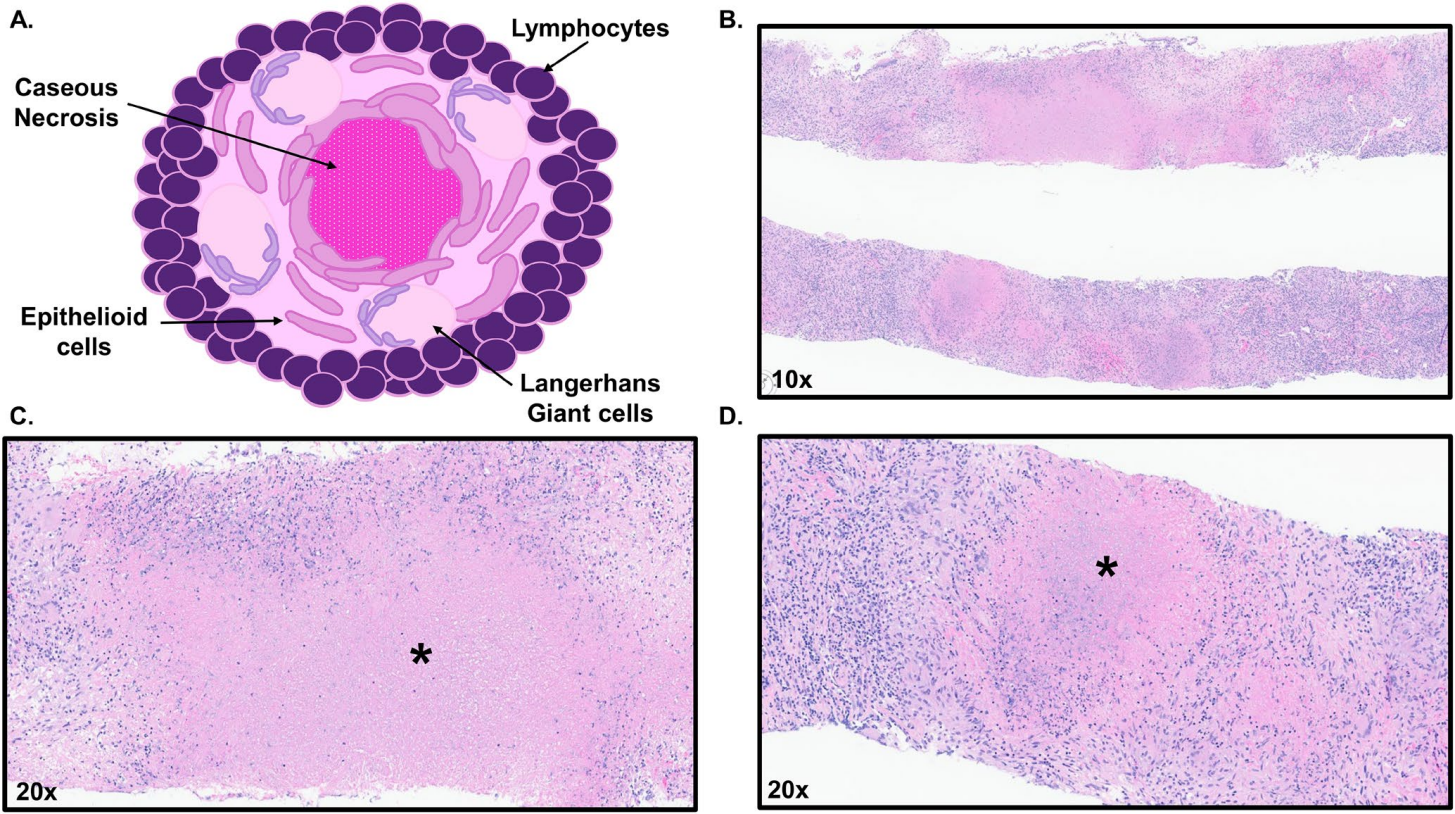
(A) Depiction of a caseous granuloma, which includes a central necrotic core enveloped by epithelioid cells and Langerhans giant cells, all of which are encased by a lymphocytic capsule. (B) H&E stain of lung biopsy at 10× magnification. (C and D) Chronic necrotic granulomatous core (*) surrounded by epithelioid and Langerhans giant cells with lymphocytic encapsulation at 20× magnification.

**FIGURE 4:**
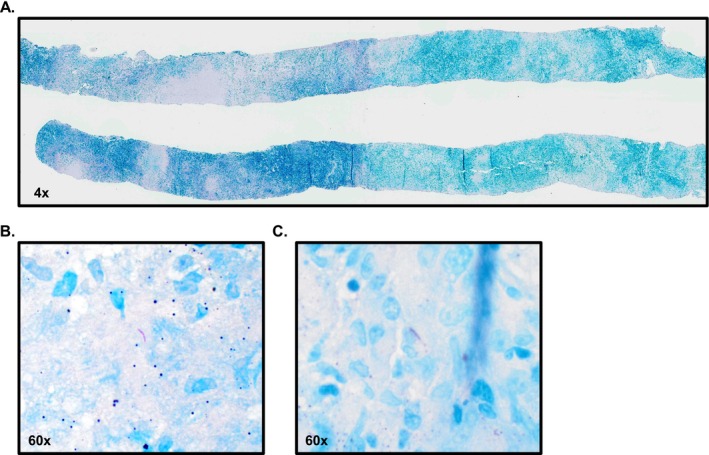
(A) AFB stain of lung biopsy at 4× magnification. (B and C) Positive identification of two AFB organisms at 60× magnification.

Per this institution's county guidelines, ruling out TB requires three independent negative bronchial sputum cultures. Additionally, acid‐fast bacilli (AFB) smear and MTB polymerase chain reaction (PCR) testing were conducted due to rapidity of results. The county guidelines state that if any component of testing is positive, the patient is deemed to have active TB. In our patient, two of the three bronchial sputum samples and the lung biopsy had positive AFB cultures. Two of the three bronchial sputum samples also had positive AFB smears and PCR. Histology of the lung biopsy indicated the presence of granulomatous inflammation, characterized by Langerhans‐type multinucleated giant cells and widespread areas of caseous necrosis on H&E stain (Figure 3A–D), and an AFB stain was positive (Figure 4A–C). Once the patient was identified as having active pulmonary TB, anti‐TB treatment was started [Rifampin 450 mg daily, Isoniazid 200 mg daily with Vitamin B6 supplementation (50 mg daily), Pyrazinamide (1000 mg daily), and Ethambutol (800 mg daily)]. The patient remained in the isolation unit for the initial five days of treatment at which point she was discharged.

## 3 Conclusion

Correct identification of an SPN secondary to an infectious source enabled appropriate rule out of a neoplastic etiology, avoiding inappropriate surgical and chemotherapeutic intervention and ensuring initiation of antimicrobial treatment and adequate community surveillance and testing.

## 4 Discussion

This case report highlights the continued diagnostic challenge in accurately distinguishing pulmonary TB from lung cancer. Overlapping, nonspecific clinical presentation and radiographic findings increase the rate of misdiagnosis [18]. Since 2011, the WHO has recommended a 4‐symptom screening tool to help rule out active TB: *absence* of chronic cough, fever, weight loss, and night sweats [19]. However, this screening tool was initially intended for patients with HIV [20] with unclear screening accuracy in the general population [21]. Some estimates suggest greater than 50% of active TB cases are missed with this screening approach due to high subjectivity [21]. Alternatively, CXR is a recommended screening alternative, but it is impractical in low‐resource areas and is associated with a low positive predictive value in deducing a lesion as TB versus alternative etiologies [22].

**FIGURE 5:**
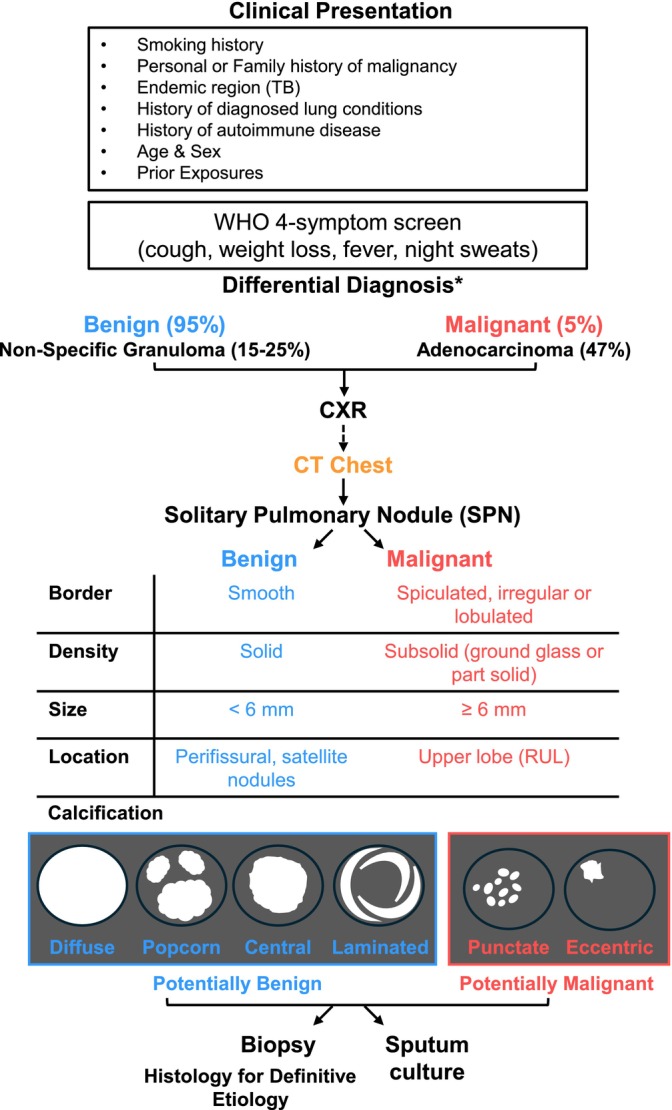
Evaluation of solitary pulmonary nodule (SPN). Patients typically have a non‐distinct clinical presentation or are asymptomatic. The WHO 4‐symptom screening criteria is typically utilized in conjunction with chest x‐ray, but both have limitations in diagnostic accuracy in TB detection. The CT chest is the gold standard for radiographic detection of SPN, given their small size (< 3 cm). However imaging, alone, is unable to diagnosis an SPN as benign versus malignant. More time‐intensive approaches, such as sputum culture or invasive procedures, such as biopsy, are necessary for definitive diagnosis. *Please see common differential diagnoses in Figure 6.

**FIGURE 6:**
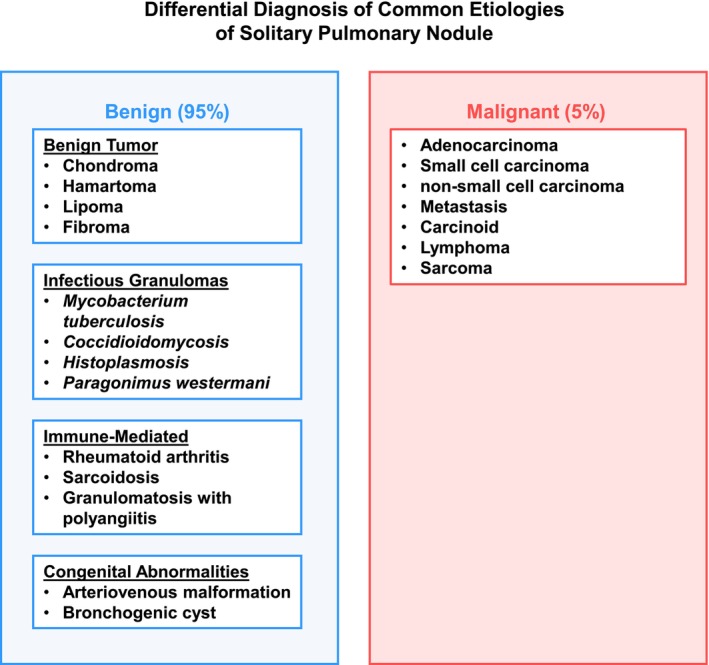
Differential diagnosis of solitary pulmonary nodule.

Atypical imaging, often characterized by detection of an SPN, is seen in approximately a third of patients with TB [23]. Further complicating correct diagnosis is the presence of a pulmonary tuberculoma, which is characterized as a well‐circumscribed nodule, and is a rare manifestation of pulmonary TB, accounting for only 9% of all cases, but is the most common type of benign SPN [24, 25]. Computed tomography (CT) remains the gold standard for SPN sensitivity detection given their small size (< 3 cm) but is unable to provide definitive diagnosis [26]. Nor is metabolic imaging able to distinguish benign vs. malignant SPNs. For example, fluorodeoxyglucose, an analog of glucose utilized in Positron Emission Tomography (PET) avidity on nuclear medicine scans to visualize and measure metabolic activity in the body, also shows increased uptake in both lung cancer and TB due to the proinflammatory and immune responses that each etiology elicits [18]. Together, lack of a definitive constellation of clinical and radiographic findings highlights the diagnostic challenge clinicians face in patients presenting with a SPN (Figure 5) and the associated broad differential (Figure 6), with definitive diagnosis reliant on histological and microbiological studies.

Further muddying the clinical picture is the relative rarity of TB within the US [27]. One retrospective study conducted at M.D. Anderson Cancer Center found a small subset (1.3%) of patients presumed to have lung cancer instead had an infectious etiology, with TB accounting for 27% of these cases [28]. In contrast, studies in TB endemic regions found 25% of patients with presumed diagnosis of lung cancer to have TB [29].

It is imperative that clinicians accurately and quickly diagnose TB to ensure rapid initiation of anti‐TB therapy and prevent unnecessary, ineffective, and possibly invasive procedures [30]. QuantiFERON Gold testing (a serum measure of interferon gamma, IFN‐γ) helps assess TB exposure, but it is unable to distinguish between latent and active TB [31]. A positive bronchial sputum culture remains the gold standard to diagnose active TB, but this testing is extremely time‐consuming (~6 weeks) due to the slow growth rate of *M. tuberculosis* . Alternative approaches include MTB‐PCR (enables rapid amplification of TB pathogen‐specific DNA) on sputum or tissue samples or AFB staining, which are faster but also have higher rates of false negatives and ultimately cannot be used to exclude TB [32].

The high global disease burden of TB has led to increased efforts focused on quick, inexpensive, and less invasive diagnostic techniques. One example is serum biomarker testing, which is gaining traction as a rapid, non‐invasive approach to distinguish TB from lung cancer by identifying a unique biomolecular signature associated with active TB [33, 34]. Due to the similarities in presentation between pulmonary TB and lung cancer, it is imperative that new diagnostic tools be developed to ensure timely and appropriate treatment intervention.

## Author Contributions

**Laura Corrales‐Diaz Pomatto‐Watson:** conceptualization, writing – original draft, writing – review and editing. **Hershan Singh Johl:** writing – review and editing. **Karleen Meiklejohn:** conceptualization, data curation, supervision, writing – review and editing.

## Funding

The authors have nothing to report.

## Consent

Written informed consent was obtained from the patient prior to publication of this case report.

## Conflicts of Interest

The authors declare no conflicts of interest.

## Data Availability

Data available on request from the authors.
